# Exercise alters the mitochondrial proteostasis and induces the mitonuclear imbalance and UPR^mt^ in the hypothalamus of mice

**DOI:** 10.1038/s41598-021-82352-8

**Published:** 2021-02-15

**Authors:** Renata R. Braga, Barbara M. Crisol, Rafael S. Brícola, Marcella R. Sant’ana, Susana C. B. R. Nakandakari, Suleyma O. Costa, Patrícia O. Prada, Adelino S. R. da Silva, Leandro P. Moura, José R. Pauli, Dennys E. Cintra, Eduardo R. Ropelle

**Affiliations:** 1 Laboratory of Molecular Biology of Exercise (LaBMEx), School of Applied Sciences, University of Campinas (UNICAMP), Limeira, SP 13484-350 Brazil; 2 Laboratory of Nutritional Genomics (LabGeN), School of Applied Sciences, University of Campinas, Limeira, SP 13484-350 Brazil; 3 Laboratory of Metabolic Disorders, School of Applied Sciences, University of Campinas, Campinas, Brazil; 4 Laboratory of Molecular Research in Obesity (Labimo), School of Applied Sciences, University of Campinas (UNICAMP), Limeira, SP 13484-350 Brazil; 5 Postgraduate Program in Rehabilitation and Functional Performance, Ribeirão Preto Medical School, School of Physical Education and Sport of Ribeirão Preto, University of São Paulo, Ribeirão Preto, SP 14040-900 Brazil; 6 CEPECE - Center of Research in Sport Sciences, School of Applied Sciences, University of Campinas (UNICAMP), Limeira, SP 13484-350 Brazil; 7 Exercise Cell Biology Lab (ECeBiL), School of Applied Sciences, University of Campinas (UNICAMP), Limeira, SP 13484-350 Brazil; 8 Department of Internal Medicine, Faculty of Medical Sciences, University of Campinas (UNICAMP), Campinas, SP 13083-872 Brazil; 9 Obesity and Comorbidities Research Center (OCRC), University of Campinas, São Paulo, Brazil

**Keywords:** Molecular biology, Protein folding, Neuroscience, Feeding behaviour

## Abstract

The maintenance of mitochondrial activity in hypothalamic neurons is determinant to the control of energy homeostasis in mammals. Disturbs in the mitochondrial proteostasis can trigger the mitonuclear imbalance and mitochondrial unfolded protein response (UPR^mt^) to guarantee the mitochondrial integrity and function. However, the role of mitonuclear imbalance and UPR^mt^ in hypothalamic cells are unclear. Combining the transcriptomic analyses from BXD mice database and in vivo experiments, we demonstrated that physical training alters the mitochondrial proteostasis in the hypothalamus of C57BL/6J mice. This physical training elicited the mitonuclear protein imbalance, increasing the mtCO-1/Atp5a ratio, which was accompanied by high levels of UPR^mt^ markers in the hypothalamus. Also, physical training increased the maximum mitochondrial respiratory capacity in the brain. Interestingly, the transcriptomic analysis across several strains of the isogenic BXD mice revealed that hypothalamic mitochondrial DNA-encoded genes were negatively correlated with body weight and several genes related to the orexigenic response. As expected, physical training reduced body weight and food intake. Interestingly, we found an abundance of mt-CO1, a mitochondrial DNA-encoded protein, in NPY-producing neurons in the lateral hypothalamus nucleus of exercised mice. Collectively, our data demonstrated that physical training altered the mitochondrial proteostasis and induced the mitonuclear protein imbalance and UPR^mt^ in hypothalamic cells.

## Introduction

The hypothalamus is responsible for the control of energy homeostasis^1^. The mitochondrial function in selective hypothalamic neurons plays a critical role in the control of food consumption, energy expenditure, and adiposity^1–4^. The loss of mitochondrial function in pro-opiomelanocortin-(POMC) and in neuropeptide Y-(NPY) producing neurons drives to abnormal energy balance^1–4^.

Mitochondrial proteins are frequently exposed to a diversity of cellular stresses that may alter the mitochondrial density and functionality^5^. Alteration in the mitochondrial proteostasis can trigger an imbalance between the mitochondrial and nuclear genomes. The stoichiometric imbalance between OXPHOS subunits encoded by nuclear DNA (nDNA) and mitochondrial DNA (mtDNA) is a conserved mechanism across the species associated with the mitochondrial improvement and longevity^6–8^. This condition is called mitonuclear imbalance and appears as a consequence of cellular stress, including caloric restriction, increased levels of reactive oxygen species, and other conditions^8^.

To adapt to the stress, eukaryotic cells, especially mitochondria, have developed over time a protein quality control system that maintains proteostasis and mitochondrial function in response to different levels of proteotoxic damage. This system is known as mitochondrial unfolded protein response (UPR^mt^) and is defined as a transcriptional response adaptive to stress, which purpose is to elevate the expression of chaperones and proteases, including the Heat Shock Protein 60 (HSP60), Lon Peptidase 1 (Lonp1), ATP-dependent Clp protease proteolytic subunit 1 (CLpP1) and YME1-Like Protein 1 (YME1L1) and others, being perceived by the cell and signalled to the nucleus with the main goal of ensuring the integrity and function of mitochondrial proteins^9^. The UPRmt improves the folding capacity of proteins, besides increasing the degradation of malformed proteins, avoiding the aggregation of non-functional proteins induced by the stress^10,11^.

One of the most potent physiological stimulators of mitochondrial biogenesis and function is the physical exercise^12,13^. Interestingly, the improvement of mitochondrial metabolism in response to exercise occurs in multiple tissues, including skeletal muscle^14–17^, liver^18^, and also some brain areas, as the hypothalamus^19,20^. Also, exercise induces an anti-inflammatory response, improves insulin and leptin sensitivity, recovering the anorexigenic and orexigenic signals in hypothalamic neurons of obese rodents^21–23^. Here, we employed a bioinformatics analysis by using a large panel of isogenic BXD mice and aerobic physical training to evaluate the role of hypothalamic mtDNA and mitochondrial proteostasis in the control of the energy homeostasis in mice. In this context, we hypothesized that physical training could induce the UPR^mt^ in the hypothalamus, improving mitochondrial function and controlling the energy homeostasis.

## Results

### The induction of hypothalamic mtDNA is associated with body weight loss

**Figure 1:**
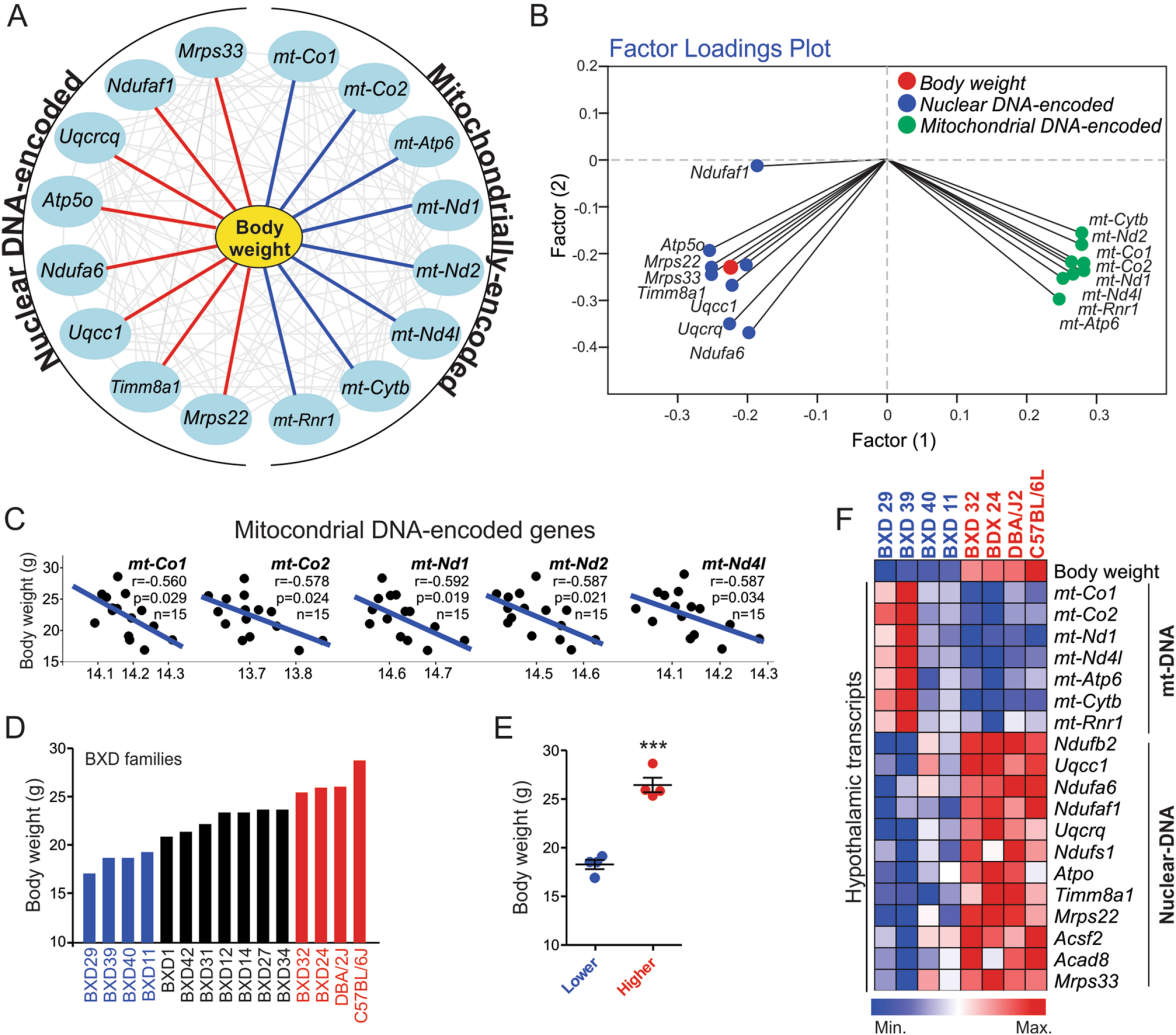
Evaluation of hypothalamic transcripts in BXD mice. (**A**) Interactome graph. Correlation positive (red line) and negative correlation (blue line) between the total body weight and mitochondrial-related genes from 15 strains of BXD mice. (**B**) Factor loading plot analysis (Pearson’s) showing the interaction between the total body weight (red) and the mitochondrial-related genes from nuclear DNA (blue) and mitochondrial DNA (green) from hypothalamic samples of BXD mice. (**C**) Pearson’s correlation between body weight and mtDNA-encoded genes (n = 15). (**D**) Body weight distribution (n = 15). (**E**) Body weight comparison between lighter and heavier strains (n = 4 per group). Student’s t-test (two-tailed). ***p < 0.001 versus control group. (**F)** Heatmap showing the gene expression of OXPHOS and other mitochondrial components in the hypothalamus (n = 4 per group). All graphs were edited in CorelDRAW 2019 software (version 21.0.0.593—https://www.coreldraw.com/en/).

To elucidate the relationship between the induction of hypothalamic mtDNA and weight loss, we performed the transcriptomic analysis using a BXD mice database. This database provides phenotypic traits and extensive evaluation of transcripts in multiple tissues, allowing an accurate assessment of traits and metabolic disorders distributed among the various isogenic strains of BXD mice^24^. The interactome analysis revealed an interesting data, showing that the body weight of BXD mice strains is negatively associated with several mitochondrial DNA-(mtDNA) encoded genes (blue lines) and positively associated with nuclear DNA-(nDNA) encoded genes (red lines) in the hypothalamus (Fig. 1A). The two-factor loadings plot and Pearson’s correlation confirmed the strong negative correlation between hypothalamic mitochondrial DNA-encoded genes and the body weight (Fig. 1B,C). To better understand these initial data, we selected the families with the lower body weight (highlighted in blue) and the families with higher body weight (highlighted in red) (Fig. 1D,E).

Interestingly, the heatmap showed that the lowest and highest cohorts, in terms of body weight, displayed an opposite pattern in mtDNA and nDNA gene signature of OXPHOS and other mitochondrial components in the hypothalamic tissue (Fig. 1F). These intriguing data suggest that the imbalance between the mtDNA/nDNA ratio in hypothalamic cells could be involved in the control of energy homeostasis. Also, the stimulation of mtDNA genes is strongly associated with the lean phenotype.

### The hypothalamic mtDNA-related genes are associated with weight loss in response to exercise

**Figure 2:**
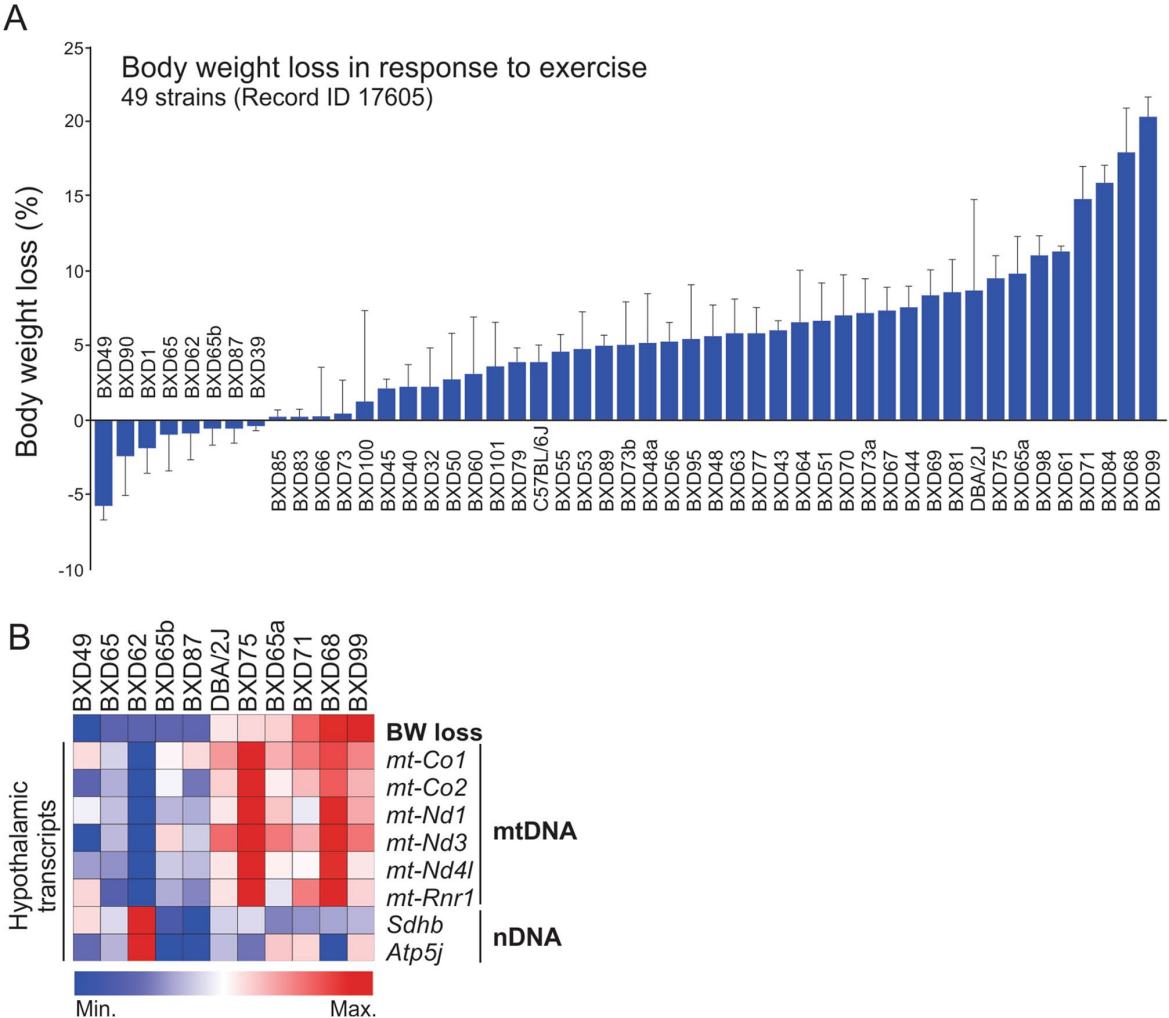
Evaluation of hypothalamic transcripts in exercised BXD cohorts. (**A**) Percentage of body weight loss in response to 3 weeks of voluntary exercise. (**B)** Hypothalamic transcripts from mitochondrial and nuclear DNA. Both graphs were edited in CorelDRAW 2019 software (version 21.0.0.593—https://www.coreldraw.com/en/).

Next, we evaluate the relationship between the hypothalamic mtDNA-encoded genes and body weight loss in response to physical exercise. Forty-nine (49) cohorts of isogenic BXD mice performed a voluntary exercise between 23 and 25 weeks of age, as previously described^25^. As expected, most of the strains presented weight loss, while some strains were resistant (Fig. 2A). Based on our initial data, we hypothesized that the pattern of hypothalamic mtDNA could be associated with the weight loss in response to exercise in these mice. After that, we monitored the mtDNA gene set from the hypothalamus of 6 specific strains that presented a consistent weight loss in response to exercise (DBA/2J, BXD75, BXD65a, BXD41, BXD68, and BXD 69). Intriguingly, these cohorts displayed high expression of mitochondrial DNA-encoded genes, including 2 OXPHOS components (*mt-Co1* and *mt-Co2*) (Fig. 2B). Collectively these data confirmed that hypothalamic mtDNA induction is closely related to body weight loss in BXD mice.

### Exercise induces hypothalamic mitonuclear protein imbalance

Based on bioinformatics data, we sought to determine the effects of physical exercise on mitochondrial proteostasis in the hypothalamus. Therefore, C57BL/6J mice were submitted to an acute session of aerobic exercise. A single session of exercise promoted a slight, but a non-substantial, augment in the expression of two mtDNA-encoded genes (*mt-Co1* and *mt-Nd1*) in the hypothalamus (Fig. S1A and B) and *Npy* expression (Fig. S1C). Thus, we hypothesized that multiple exercise sessions could be more efficient to alter the mitochondrial proteostasis in the hypothalamus.

**Figure 3:**
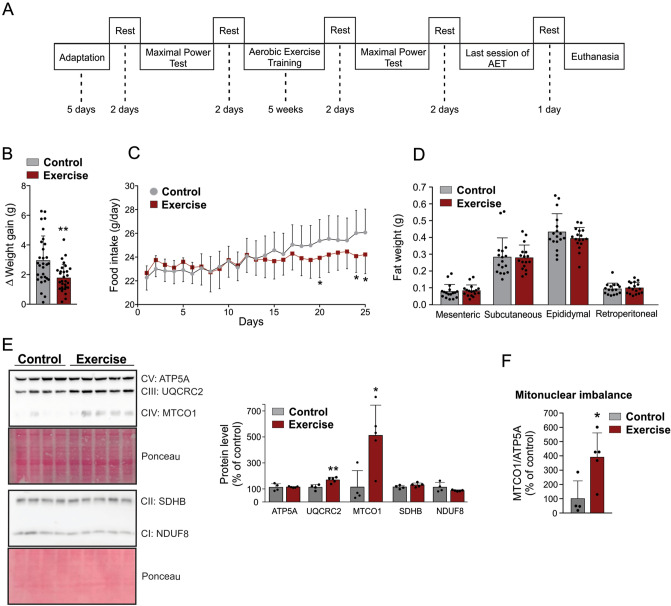
Effect of exercise on OXPHOS components and mitonuclear imbalance in hypothalamic tissue. (**A**) Experimental design. (**B**) Body weight gain (n = 30 per group). (**C**) Food intake (n = 15 per group). (**D**) Fat mass depots (n = 17 per group). (**E**) Western blot showing the oxidative phosphorylation complex components (control, n = 4, and exercise, n = 5). (**F**) mt-CO1/ATP5a ratio, for characterization of mitonuclear imbalance (control, n = 4, and exercise, n = 5). AET: aerobic physical training. “Student’s t-test (two-tailed); *p < 0.05 versus control group. **p < 0.01 versus control group. Full-length blots are presented in Supplementary Figure S4. Figures (**B**–**F**) were created in CorelDRAW 2019 software (version 21.0.0.593—https://www.coreldraw.com/en/).

Thus, we monitored the mitochondrial metabolism in the hypothalamus in response to aerobic training in C57BL/6J mice, as detailed in the experimental design (Fig. 3A). As expected, five weeks of treadmill running altered the energy balance, reducing the body weight gain (Fig. 3B and S2A) and food intake (Fig. 3C). Exercise did not change the amount of different fat depots (Fig. 3D). As expected, the aerobic exercise protocol was efficient in increasing the aerobic capacity in trained animals (Fig. S2B).

The Western blot analysis revealed that physical exercise did not change the protein content of OXPHOS components encoded by nDNA in the hypothalamus, including ATP5A (complex V), SDHB (complex II) and NDUF8 (complex I), but increased UQCRC2 (complex III) protein levels (Fig. 3E). However, exercise increased the protein content of mt-CO1 (complex IV), the OXPHOS component encoded by the mtDNA (Fig. 3E). Furthermore, we observed that physical training led to the mitonuclear protein imbalance in the hypothalamus of mice by increasing the mt-CO1/ATP5A ratio (Fig. 3F). The in vivo experiment confirmed that exercise altered the mitochondrial proteostasis in the hypothalamus of mice, which was accompanied by the modification in the energy balance.

### Physical training induces hypothalamic UPR^mt^

**Figure 4:**
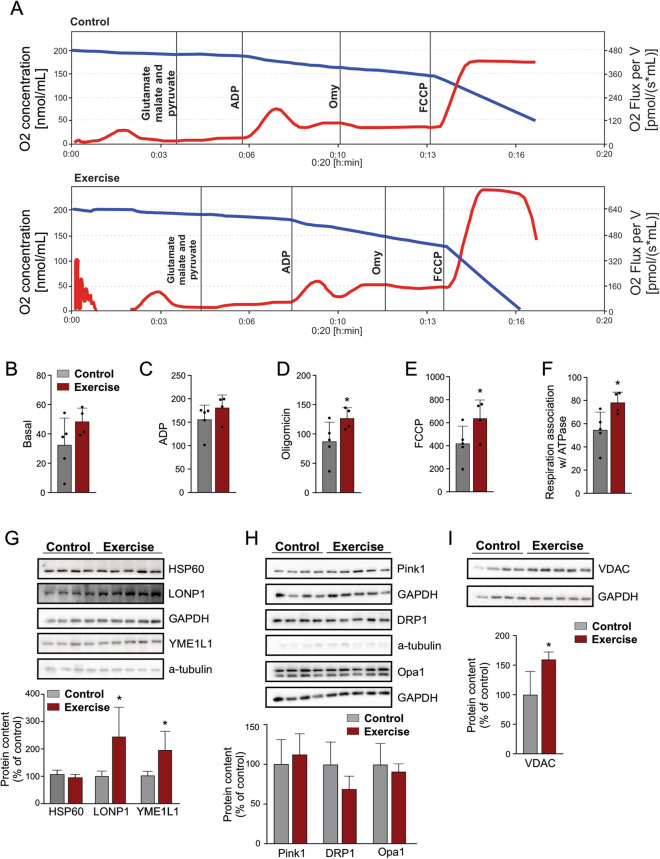
Mitochondrial markers and oxygen consumption analysis. (**A**) Linear O_2_ consumption graphs from control and exercise groups. (**B**) Basal levels of oxygen consumption. (**C**) Respiration stimuli after ADP addition. (**D**) Inhibition of mitochondrial respiration after oligomycin addition. (**E**) Maximal capacity of the electron transfer system after uncoupler FCCP addition. (**F**) Mitochondrial respiration capacity that is associated with ATP synthesis (Complex V) acquired by oligomycin values minus basal values (n = control, n = 5 and exercise, n = 4 in **B**–**F**). Western blot showing hypothalamic protein content of (**G**) UPR^mt^ markers (control, n = 4, and exercise, n = 5). (**H**) Pink1, DPR1, and Opa1 (control, n = 4, and exercise, n = 5). (**I**) VDAC (control, n = 4, and exercise, n = 5). “Student’s t-test” (one-tailed **B**–**F**; two-tailed **G**–**I**). *p < 0.05 versus control group. Full-lenth blots are presented in Supplementary Figure S4. Figures (**B**–**I**) were created in CorelDRAW 2019 software (version 21.0.0.593—https://www.coreldraw.com/en/).

Next, we determined the effects of physical training on mitochondrial function and UPR^mt^ markers in the hypothalamus. By using the mitochondrial extract from the whole brain, we determined oxygen consumption rates (Fig. 4A) and it was found that physical exercise did not change the basal mitochondrial respiration (Fig. 4B) but increased the maximum mitochondrial respiratory capacity (Fig. 4C–F). The Western blot analysis demonstrated that the regular exercise did not change the HSP60 protein content but increased the other two UPR^mt^ markers (LONP1 and YME1L1) in the hypothalamic tissue (Fig. 4G). On the other hand, our data demonstrated that five weeks of exercise were not sufficient to alter the markers of mitophagy (Pink1), mitochondrial fission (DRP1), or mitochondrial stability (Opa1) in the hypothalamus (Fig. 4H). Also, the mitonuclear imbalance and UPR^mt^ were accompanied by high protein content of the critical molecule that controls mitochondrial metabolism and function, the Voltage-Dependent Anion Channel 1 (VDAC1) in the hypothalamus of exercised mice (Fig. 4I). These data demonstrated that in response to the alteration in the mitochondrial proteostasis induced by physical exercise, the hypothalamic cells elicited the UPR^mt^ response to ensure the function and integrity of mitochondria.

### Hypothalamic mtDNA-encoded genes and orexigenic signals

**Figure 5:**
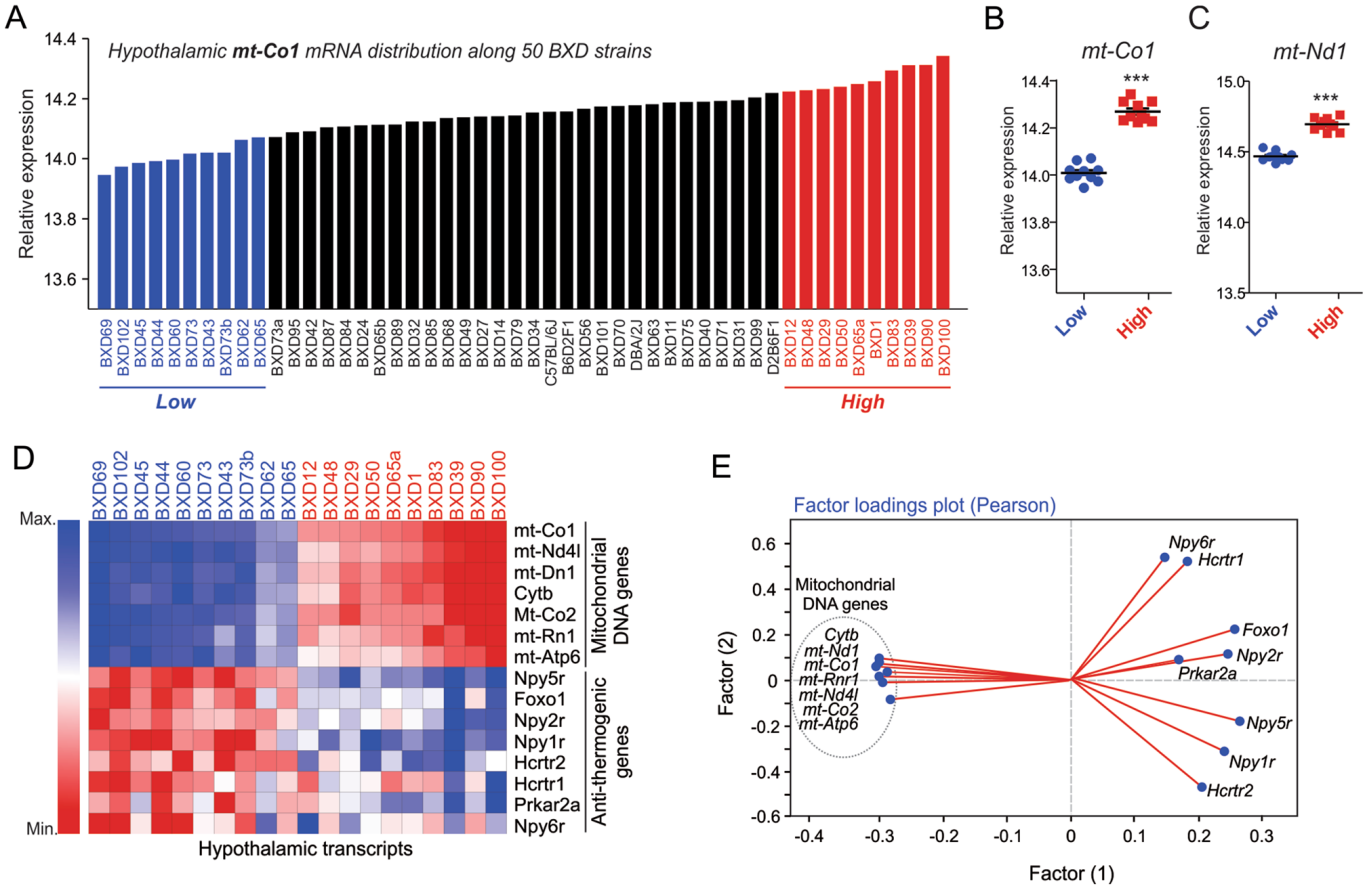
Hypothalamic mt-DNA and the orexigenic signals. (**A**) Hypothalamic *mt-Co1* mRNA distribution in BXD mice (n = 50). Comparison between lowest (blue) and highest (red) hypothalamic: (**B**) *mt-Co1* and (**C**) *mt-Nd1* mRNA values (n = 10). Student’s t-test (two-tailed). ***p < 0.001 vs control group. (**D**) Heatmap showing the gene expression from mtDNA-encoded genes and orexigenic genes. (**E**) Factor loading plot analysis (Pearson’s) showing mtDNA-encoded genes and orexigenic genes in BXD strains. All graphs were edited in CorelDRAW 2019 software (version 21.0.0.593—https://www.coreldraw.com/en/).

To better understand the relationship between the hypothalamic mtDNA-encoded genes and the control of the energy homeostasis, we turned to the BXD database. We first observed the differential pattern of *mt-Co1* mRNA in the hypothalamus of 50 strains of BXD mice (Fig. 5A). The significative difference was observed between 10 strains with lower (blue) and 10 strains with higher (red) of *mt-Co1* mRNA in the hypothalamus (Fig. 5B). These strains also displayed the same pattern for another mtDNA-encoded gene, *mt-ND1* (Fig. 5C). The heatmap analysis shows a clear negative correlation between the hypothalamic mtDNA-encoded genes and orexigenic markers, whereas 10 strains with higher mtDNA-encoded genes in the hypothalamus showed low expression of several orexigenic- and anti-thermogenic genes, including NPY receptors (*Npyr1*, *Npyr*2, *Npyr*5 and *Npyr*6), AMPK2 (*Prka2a*) FoXO1 (*Foxo1*) and orexin receptors (*Hcrtr1* and *Hcrtr2*) (Fig. 5D). The two-factor analysis confirmed that several hypothalamic genes encoded by mtDNA are strongly and inversely associated with orexigenic- and anti-thermogenic genes (Fig. 5E). These data suggest that the stimulation of mitochondrial genes from the mtDNA in hypothalamic cells is associated with the control of the energy homeostasis.

### Exercise stimulates mtCO-1 protein content in hypothalamic neurons

Due to the negative correlation between hypothalamic mtDNA-encoded genes and orexigenic neuropeptides, we next sought to investigate if the physical exercise affects the abundance of mtDNA markers in the neuronal subpopulation specifically in the lateral hypothalamus, an area related to orexigenic neuropeptides and hunger signals.

**Figure 6:**
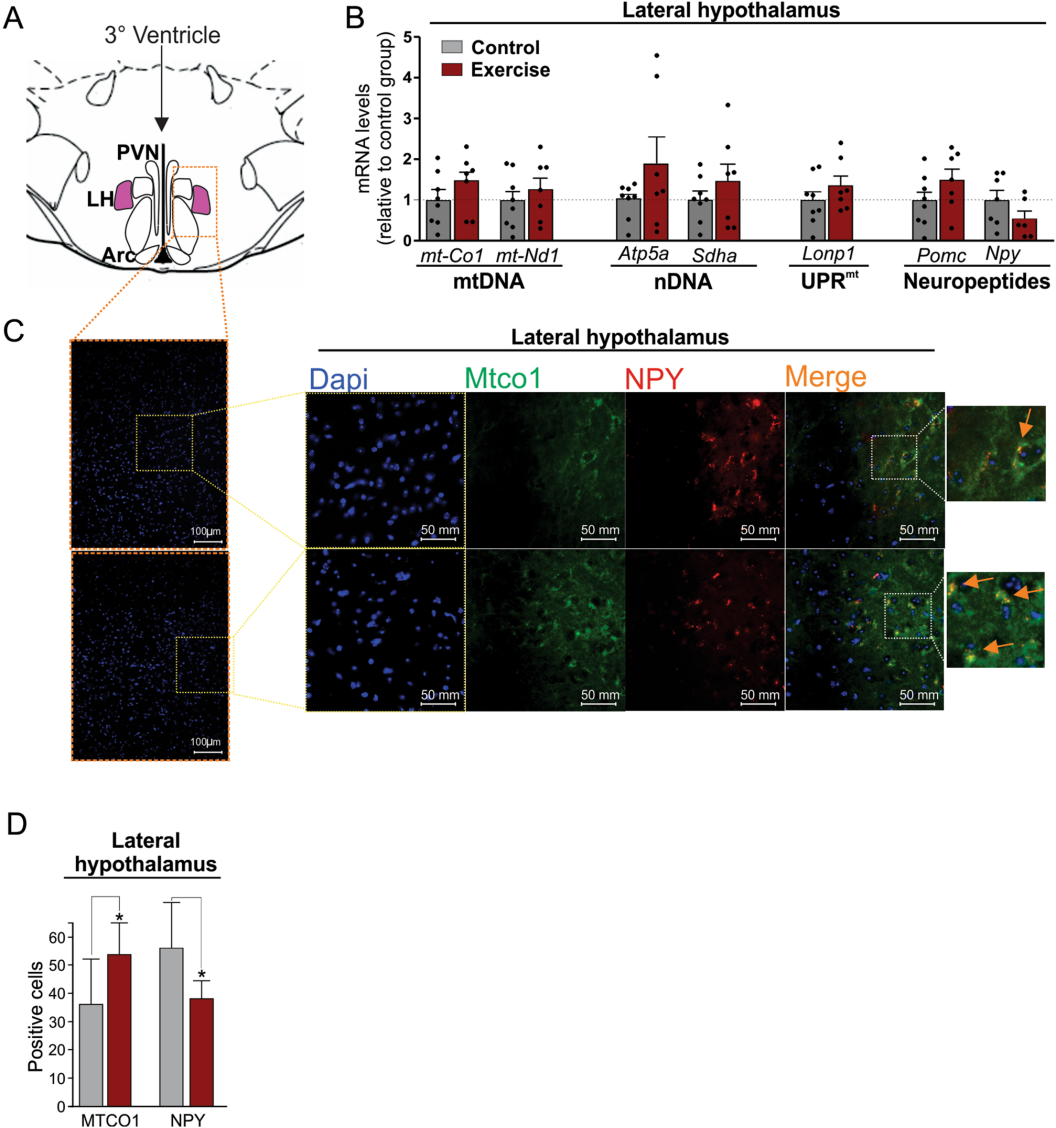
Evaluation of hypothalamic *mt-Co1* mRNA levels and orexigenic factors. (**A**) Schematic view of hypothalamic nuclei, highlighting the lateral hypothalamus. (**B**) Genes expression assessment. Student’s t-test (two-tailed). (n = 7–8 per group). (**C**) Immunofluorescence assay showing DAPI staining (200 and 400 × magnification) and the presence of mt-CO1 (green) in NPY (red) in neurons of the lateral hypothalamus nucleus of mice (400 × magnification) and the spotlighted image was acquired by using digital zoom. Orange arrows indicate mt-CO1/NPY colocalized neurons. (**D**) Quantification of mt-CO1 and NPY in the lateral hypothalamus of mice. Student’s t-test (two-tailed). *p < 0.05 versus control group (n = 3–4 per group). All figures were created in CorelDRAW 2019 software (version 21.0.0.593—https://www.coreldraw.com/en/).

The marker of mtDNA, nDNA, UPR^mt^ and neuropeptides expression were evaluated in separated hypothalamic nuclei of sedentary and trained mice. No differences were found in the arcuate and paraventricular nuclei (data not shown). Among the hypothalamic nuclei studied, the most interesting results were found in the lateral hypothalamus (Fig. 6A). We observed that exercise promoted a slight enhancement in the gene expression of OXPHOS subunits encoded by mtDNA (*mt-Co1* and *mtNd1*) and nDNA (*Atp5a* and *Sdha*), UPRmt marker (*Lonp1*) and neuropeptides (*Pomc*) in the lateral hypothalamus. On the other hand, a slight reduction of *Npy* mRNA was observed; however, we still did not find statistical differences between the groups (Fig. 6B).

To deeply understand the effects of exercise in the lateral hypothalamus, we evaluated the mt-CO1 accumulation in different neuronal subpopulations in this nucleus. The immunofluorescence staining revealed that exercise increased the mt-CO1 protein content in NPY-producing neurons in the lateral hypothalamus (Fig. 6C,D). No difference was found in mt-CO1 positive cells in Pomc-producing neurons (Fig. S3A and B). These data suggest that exercise modulates the mitochondrial proteostasis in orexigenic neurons of lateral hypothalamus.

## Discussion

Neuronal mitochondrial function plays a crucial role in the control of energy homeostasis in mammals^1,3,4,7^. The mitochondrial proteostasis perturbation triggers distinct signals to recover the mitochondrial and cellular homeostasis, such as the mitonuclear protein imbalance and UPR^mt^^6,10,11,26^. By using bioinformatics analysis and in vivo experiments, here we provide consistent data showing that physical training alters the mitochondrial proteostasis, eliciting the mitonuclear imbalance and UPR^mt^ in the hypothalamus of mice. These hypothalamic alterations were accompanied by the reduction of body weight and energy intake. Also, we demonstrated an exciting connection between the improvement of hypothalamic mitochondrial metabolism and exercise-induced body weight loss in mice.

The mitochondrial dysfunction in the central nervous system is associated with synapse loss and neuronal dysfunction^27^. Thus, the maintenance or improvement of mitochondrial functionality in the brain could be essential for several organic functions and prevents neurological disorders. An elegant study reported that Nicotinamide Riboside (NR), an NAD^+^ precursor, recapitulated the mitochondrial proteostasis by increasing the UPR^mt^, reducing amyloid-β proteotoxicity in mouse and worm Alzheimer’s disease models^28^. Also, some studies found hypothalamic mitochondrial abnormalities affecting food intake in mice fed with high fat-diet (HFD)^29,30^ and a novel role of POMC in feeding by cannabinoids stimuli that are mediated by alterations in mitochondrial function^31^.

In terms of energy homeostasis, the mitochondrial dysfunction in pro-opiomelanocortin (POMC)-producing neurons induces poor energy balance leading to body weight gain and metabolic dysfunction^3,32,33^. A recent study presented that the ablation of mitofusin 1, specifically in POMC-producing neurons, resulted in defective mitochondrial function and altered glucose sensing in mice^33^. Furthermore, mice lacking mitofusin 2 in POMC-producing neurons also displayed mitochondrial abnormalities, hypothalamic leptin resistance, and obesity^3^. Here, we found no difference of mt-CO1 accumulation in Pomc neurons, however, exercise stimulated the OXPHOS component from mtDNA in NPY-producing neurons in the lateral hypothalamus and reduced the food consumption and weight gain in mice. Collectively these studies strongly suggest that the neuronal mitochondrial activity plays a critical role in the control of energy homeostasis.

Interestingly, we found that physical training enhanced the VDAC1 protein content in mice hypothalamus. VDAC is an outer mitochondria membrane protein, which is involved in several mitochondrial functions including, transport of metabolites, energy production, and apoptosis^34,35^. In parallel, we also observed that exercise increased the mitochondrial respiration in the central nervous system, which leads to a better organelle function for the exercise group.

The mitonuclear protein imbalance is critical for the UPR^mt^ activation in worms and mammals^26^. These mechanisms seem to be essential to maintain nDNA- and mtDNA-encoded OXPHOS mitonuclear imbalance stimulation, increasing the mitochondrial function and improving the metabolic aspects in hepatic tissue^36^, skeletal muscle of mice^14,17^ and the skeletal muscle of type 2 diabetic patients^37^. Beyond the peripheral tissues, the present study showed that physical stress stimulates the mitonuclear imbalance in hypothalamic cells. Interestingly, we found a discrepant signature of nDNA- and mtDNA-encoded OXPHOS gene expression in the hypothalamus of a large panel of isogenic BXD mice strains, whereas the lighter strains displayed higher mtDNA-encoded OXPHOS gene expression in the hypothalamus when compared to the heavier ones. Importantly, when we used the mitochondrial cocktail antibody to evaluate the OXPHOS protein components, we found an increase in mtCO1 protein content in the hypothalamus of mice, while no differences were observed in SDHD and NDUF8 (nDNA-encoded proteins). These findings reaffirm what we found in bioinformatics analysis regarding pattern of mtDNA encoded genes expression in response to exercise and body weight loss.

Strikingly, the immunofluorescence assay showed a high level of mt-CO1 protein content in NPY-producing neurons of trained mice and reduced the expression of this orexigenic neuron in the lateral hypothalamus, which is an area linked with feeding behaviour responsible for signals to increase food intake^38^. The stimulation of mtDNA, specifically in NPY-producing neurons in the lateral hypothalamus, can contribute to the suppression of orexigenic signals, which is in accordance with the food intake reduction observed in exercised animals in Fig. 3C. Also, increased mitochondrial protein content is linked to better activity of neurons, which demonstrate that the neuron is functioning properly^39^. In the same line, the bioinformatics data confirmed that several BXD mice strain with high levels of *mt-Co1* mRNA in the hypothalamus displayed low levels of anti-thermogenic and orexigenic genes in this tissue. Thus, this is the first evidence that neuronal mitonuclear imbalance is involved in the control of energy homeostasis in mammals.

Our data showed that, at least in lean mice, physical training did not change the mitochondrial dynamic markers such as Pink1, DRP1, and Opa1. These data corroborate a previous study showing that three weeks of physical training has augmented DRP1 protein in aged, but not in young mice brain^40^. On the other hand, we found the augment of Lonp1 and YME1L1 protein content, recognized as UPR^mt^ components^41,42^. Lon-peptidase 1 (Lonp1) functions as a mediator to selective degradation of misfolded proteins in the mitochondrial matrix and preserves mitochondrial integration^43^. Interestingly, a study on Lonp1 inactivation in *Drosophila* detected that higher levels of this protein lead to reduced mitochondrial protein translation^41^ to avoid the overload in the system. Also, this protein can behave as a chaperone, ameliorating this stressed scenario inside the cell. YME1-Like Protein 1 (YME1L1) is described as a selective folded and unfolded protein degradation stimulant, and the degradation of this protein reduces mitochondrial capacity during oxidative stress^42^. In yeast, the YME1L1 homologous (Yme1p) has been shown to degrade nonassembled mitochondrial proteins^44^. In our experimental model, we found that physical training induced YME1L1 and Lonp1 accumulation but did not change HSP60 content. This result indicates that the mechanism responsible for the protection and amelioration of the mitochondrial metabolism in response to stress is activated.

The stress sensing signalling could mediate the UPR^mt^ stimulation in response to exercise. Importantly, it has been shown that JNK signalling stimulates UPR^mt^-related genes^45^. Recently, Kwon and colleagues demonstrated that endurance exercise increased JNK^Thr183/Tyr185^ phosphorylation, which was associated with the improvement of the mitochondrial dynamic in the mouse brain^46^. However, the mechanism by which exercise induces the UPR^mt^ in the hypothalamus remains unclear and deserves further investigation.

In summary, our study demonstrated that exercise alters the mitochondrial proteostasis and elicits the mitonuclear imbalance and some UPR^mt^ markers in the hypothalamus of mice, stimulating the OXPHOS component from mtDNA in NPY-producing neurons in the lateral hypothalamus of mice. Thus, the stimulation of mitochondrial genes from the mtDNA in hypothalamic neurons through physical exercise could be an attractive therapeutic target to improve the mechanisms involved in the control of energy homeostasis.

## Material and methods

### Animals and housing

Eight-week-old male C57BL/6J mice, weighting (20 g ± 2 g) were used from the University of Campinas (UNICAMP) animal facility. This specie and model was chosen for being a homogeneous line of animals. The animals were placed in groups of five per cage, with controlled temperature (between 20 and 22 °C) on a 12:12 h light–dark cycle with food, diet from AIN-93^47^ prepared at the School of Applied Sciences of UNICAMP, and water ad libitum. Mice were randomly distributed in the control and exercise groups. The ethics committee of UNICAMP approved the experiments, protocol number 4892-1/2018. The number of animals used in each experiment is specified in the figure legends. All methods were carried out in accordance with the ARRIVE checklist^48^.

### Incremental exercise test

Mice were submitted to incremental maximal power test on the first day of the intervention, as previously described^49^, to determine the mice exhaustion and individual workloads. We used the exhaustion information of each mouse to calculate an intensity corresponding to the 60% peak workload and to use in continuous training. After, animals were randomly divided into chronic and acute exercise groups. After the implementation of chronic exercise protocol, the animals were submitted to the same test again to confirm the improvement in the aerobic performance.

### Acute exercise protocol

The acute exercise group was submitted to one session of training, with one hour of duration, on a motor treadmill at 60% of workload peak. Mice were adapted to the treadmill for 10 min at 6 m/min for five days^49^, aiming to minimize the stress induced by the equipment. Animals were anesthetized 2 h after the exercise session for hypothalamus removal.

### Chronic exercise protocol

The chronic exercise group was submitted to 5 weeks of training, with 1 h of duration 5 days per week, on a motor treadmill at 60% of workload peak. Mice were adapted to the treadmill for 10 min at 6 m/min for five days^49^, aiming to minimize the stress induced by the equipment. The animal’s welfare and stress signs were monitored daily before, during, and after exercise sessions, with the control group included. Animals were anesthetized 24 h after the last exercise session for hypothalamus removal.

### Tissue sampling

Mice were anesthetized with an intraperitoneal injection of chlorohydrate of ketamine (200 mg/kg, Parke-Davis, Ann Arbor, USA) and xylazine (16 mg/kg, Rompun, Bayer, Leverkusen). The hypothalamus tissue was homogenized in extraction buffer (Triton X 100 1%, Tris 100 mM–pH 7.4, sodium pyrophosphate 100 mM, sodium fluoride 100 mM, EDTA 10 mM, sodium orthovanadate 10 Mm, PMSF 2 Mm, aprotinin 0.1 mg/mL at 4 °C; Sigma-Aldrich, Saint Louis, MO, USA) and the samples were stored at − 80 °C as previously described^50^. Protein concentration was determined by the Bicinchoninic Acid (BCA) Method. Laemmli buffer^51^ was added to each of the samples, which were stored at − 80 °C for western blot analyses.

### Lateral hypothalamus dissection

Lateral hypothalamus (LH) samples were quickly dissected in a stainless steel matrix with razor blades as described previously^52^ and frozen in liquid nitrogen for further gene expression evaluation.

### Western blotting

The western blot was performed as previously described^53^. Anti-OXPHOS (Abcam Plc, Cambridge, United Kingdom; Cat# ab110413, RRID: AB_2629281), anti-HSP60 (Santa Cruz Biotechnology, CA, USA; Cat# sc-13115, RRID: AB_627758), anti-YME1L1 (Proteintech, Rosemont, Illinois, USA; Cat# 11510-1-AP, RRID: AB_2217459), anti-PINK1 (Proteintech, Rosemont, Illinois, USA; 23274-1-AP), anti-OPA1 (Cell Signaling Technology; Cat# 80471, RRID: AB_2734117), anti-VDAC (Cell Signaling Technology; Cat# 4866, RRID: AB_2272627), anti-DRP1 (Cell Signaling Technology; Cat# 8570, RRID: AB_10950498), anti-α-tubulin (Cell Signaling Technology; Cat# 2144, RRID: AB_2210548) and anti-GAPDH (Cell Signaling Technology; Cat# 2118, RRID: AB_561053), anti-LONP1 (Bioss Antibodies, Boston, Massachusetts, USA; Cat# bs-4245R, RRID: AB_11051909). All antibodies were used at dilution 1:1000. Ponceau was from Sigma-Aldrich (Saint Louis, MO, USA). The respective loading control normalized the western blotting results, and the final values were given in the percentage of the respective control group.

### High-resolution respirometry

To determine oxygen consumption rates with Oxygraph-2k (Oroboros Instruments, Innsbruck, Austria), the mitochondria were isolated from mouse brain^54^. Therefore, dissected brain was washed in ice-cold MIR05 (110 mM sucrose, 60 mM K-lactobionate, 0.5 mM EGTA, 3 mM MgCl_2_, 20 mM taurine, 10 mM KH_2_PO_4_, 20 mM HEPES, pH 7.1 at 30 °C, and 0.1% BSA) and brain wet-weight was measured. The tissue was cut with a small scissor and suspended in five times the tissue wet-weight in MIR05 and was homogenized using a Potter homogenizer with 8–10 strokes at 1000×*g*. The homogenate was transferred to a falcon containing 20 mL of an isolation buffer (320 mM sucrose, 1 mM EDTA, 10 mM TRIS, pH 7.4 with HCl) and centrifuged for 10 min at 1000×*g*, 4 °C. Then, the supernatant was transferred to a new tube and centrifuged for 10 min at 6200×*g*, 4 °C. The supernatant was discarded, and the pellet was gently washed with ice-cold isolation buffer. The pallet was resuspended in approximately 200 µL of MIR05. To measure mitochondrial complex I-dependent respiration, ADP (2 mM), pyruvate (5 mM), malate (2 mM), and glutamate (20 mM) were added to mitochondria in respiration buffer MIR05. Combined complex I and complex II respiration was assessed by the addition of succinate (10 mM). Subsequently, mitochondrial coupling was evaluated by the inhibition of ATP synthase by adding oligomycin (1.5 mg/mL) and uncoupling by a multiple-step carbonylcyanide p-trifluoromethoxyphenyl-hydrazone (FCCP) titration.

### RT-qPCR

RNA was extracted from the separated hypothalamic nucleus of mice using the RNeasy Micro Kit (Qiagen, Courtaboeuf, France). RNA was reverse transcribed using the qMax First Strand cDNA Synthesis Flex Kit (Accuris Instruments, Edison, NJ, USA). Quantitative real-time PCR was performed by using PowerUp SYBR Green Master Mix (Applied Biosystems, Foster-City, CA, USA) in Applied Biosystems 7500 Real-Time PCR systems. Relative mRNA expression was determined by the ΔΔCt method and normalized to GAPDH. The primers sequences utilized are available in the supplementary material (Supplementary Table 1).

### Immunofluorescence analysis

Mice destined for histological analysis by immunofluorescence were previously anesthetized and fixed with 4% paraformaldehyde solution. After fixation, brains were collected and immersed in the same fixative solution for 24 h at 4 °C. Afterward, brains were cryopreserved in 30% sucrose solution, and coronal sections (15 μm) were performed with the support of the cryostat (Leica CM1850). The sections were incubated in a blocking solution (1 × TBST with 3% BSA) and subsequently incubated overnight with primary antibodies of interest at the 1: 100 dilutions of mt-CO1 (bs-3953R; Bioss Antibodies, Boston, Massachusetts, USA) and NPY (sc-14728; Santa Cruz Biotechnology, CA, USA) and at dilution of 1: 2000 of POMC (H-029-30; Phoenix Pharmaceuticals, Burlingame, CA, USA). Finally, the sections were incubated with IgG secondary antibodies conjugated with FITC green fluorophore (1: 100; Santa Cruz Biotechnology, CA, USA) or red rhodamine fluorophore (1: 200; Santa Cruz Biotechnology, CA, USA). Finally, the cuts were prepared with VECTASHIELD Antifade Mounting Media with DAPI (# H-1200; Vector Laboratories, Burlingame, CA, USA), staining the nucleus with a blue colour. Images were acquired by Leica Application Suite software. A quantitative assessment was accomplished by one experienced observer-blind counting the number of positively stained cells (red + DAPI, green + DAPI, or Merged + DAPI). Each image (12 × 9 cm) containing a separated hypothalamic nucleus from the left and right side was gridded in 108 squares, equal-sized field (1 × 1 cm). Each square was manually counted by using the CorelDRAW 2019 software (version 21.0.0.593). The counting was performed through one picture of each area per animal, totalizing five sections of each area of the arcuate nucleus, lateral hypothalamus, and paraventricular nucleus. The images were achieved at a magnification of 200 × and the spotlighted image was acquired in digital zoom of 400 ×.

### Bioinformatics analysis

Bioinformatics analyses were performed using a database accessible on Genenetwork (http://www.genenetwork.org). Hypothalamic transcripts were analysed from several families of BXD mice (Hypothalamus Affy MoGene 1.0 ST (Nov10)), as previously described^24^. The interactome and the two factors analyse graphs were generated on Genenetwork. After that, the graphs were edited in Corel DRAW 2019 software (version 21.0.0.593) obtained on (https://www.coreldraw.com/en/). GraphPad Prism 7 was used for the Pearson or Spearman’s correlations. Gene-E online software was assessed on (https://software.broadinstitute.org/GENE-E/) for the heat map analyses. The hypothalamic mRNA levels or phenotypic features of individual animal are accessible in the supplementary files (Supplementary Tables 2–4).

### Statistics

All results were expressed as a mean ± standard error from the mean (SEM). The data were analyzed by “t-Student” or “Mann–Whitney U test”, according to the normality test of Shapiro–Wilk. The statistical significance used was *p* < 0.05. The Pearson or Spearman’s correlations were performed using GraphPad Prism 7 and images were created by CorelDRAW 2019 (version 21.0.0.593).

## Supplementary Information

Supplementary Information.
