# Supplementary material for: Exercise alters the mitochondrial proteostasis and induces the mitonuclear imbalance and UPRmt in the hypothalamus of mice
Source: Sci Rep. 2021 Feb 15;11:3813. doi: 10.1038/s41598-021-82352-8 (PMC7884690; doi:10.1038/s41598-021-82352-8)
Supplement: Supplementary file 1 — Supplementary Information. [file 41598_2021_82352_MOESM1_ESM.pdf]

Exercise Alters the Mitochondrial Proteostasis and Induces the Mitonuclear Imbalance and UPR^{mt} in the Hypothalamus of Mice

Renata R. Braga[1], Barbara M. Crisol[1], Rafael S. Brícola[1], Marcella R. Sant'ana[2], Susana C. B. R. Nakandakari[2], Suleyma O. Costa[3], Patrícia O. Prada[4], Adelino S. R. da Silva[5], Leandro P. Moura[1,6,7], José R. Pauli[1,6] Dennys E. Cintra[2] and Eduardo R. Ropelle*[1,5,8,9]

Supplementary Material

Figure S1

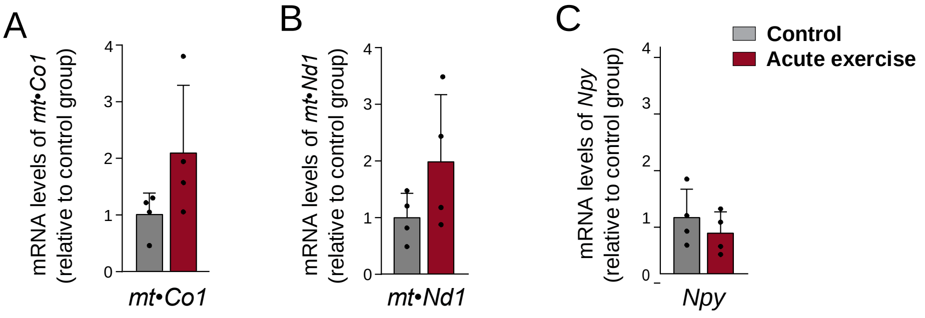

Figure S1. Effect of acute exercise on **A.** *mtCo1* **B.** *mt•Nd1* **and C.** *Npy* mRNA in the hypothalamus (n=4 per group). "Student's t-test (two•tailed).

Figure S2

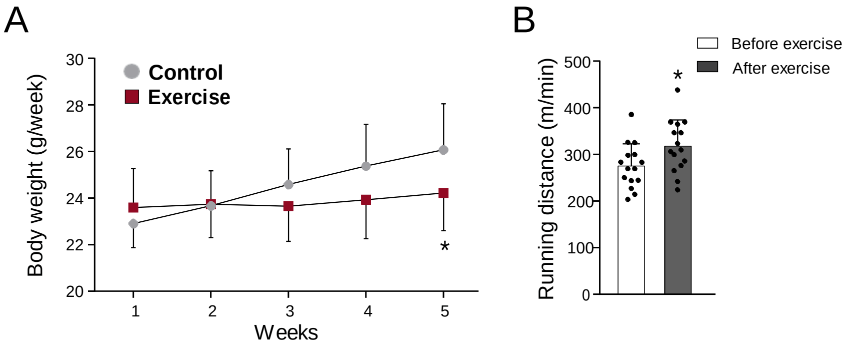

Figure S2. Effect of chronic exercise on A Body weight gain (n=15 per group). B. Total running distance (n=15 per group). “Student’s t-test (two-tailed); *p<0.05 vs control group.

Figure S3

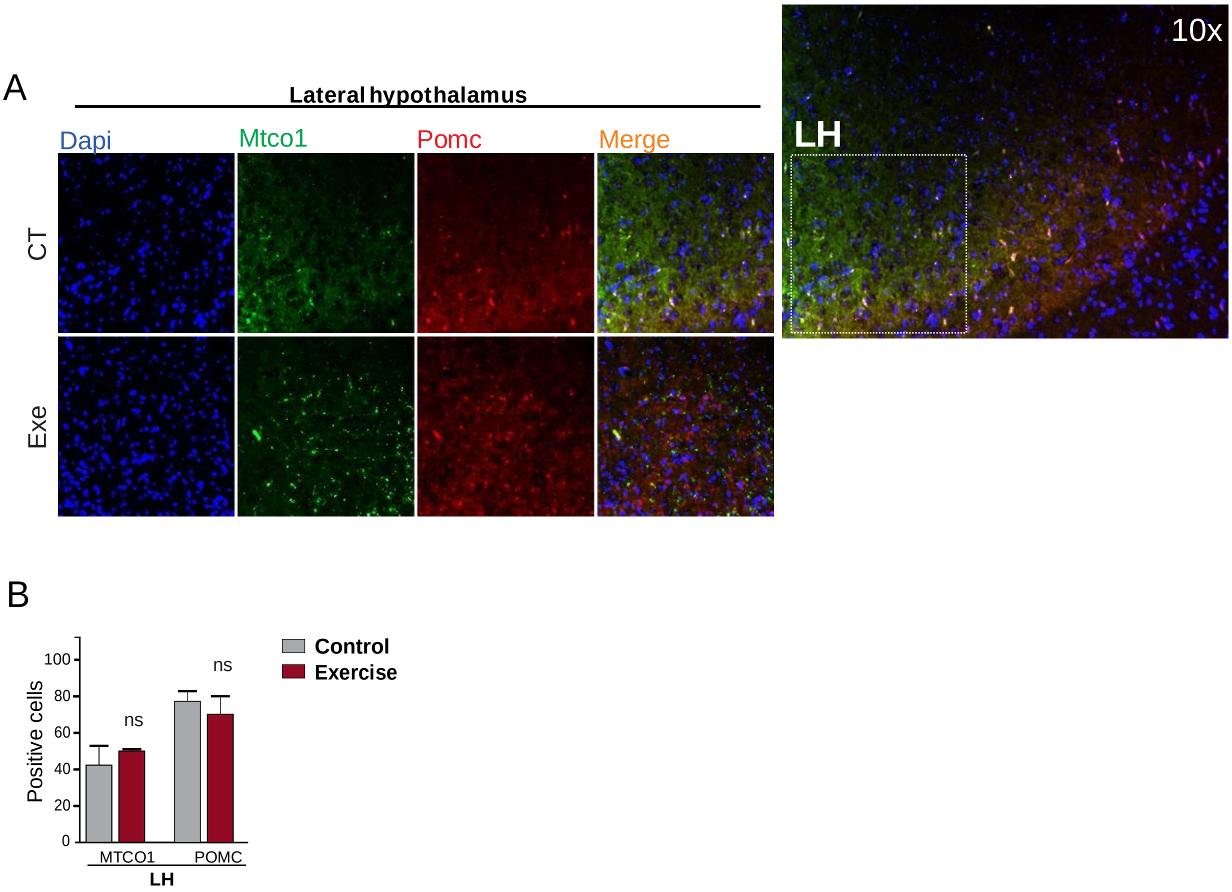

Figure S3. Evaluation of hypothalamic mtCO1 levels and anorexigenic factors. A. Immunofluorescence assay showing the presence of mtCO1 (green) in POMC (red) in neurons of the lateral hypothalamus nucleus of mice (400 x magnification). Orange arrows indicate mtCO1 and / or POMC-labeled neurons. B. Quantification of mtCO1 and POMC in the lateral hypothalamus of mice (n= 3 CT x 2 EX per group).

Figure S4

Figure 3E - Membranes and ponceau staining

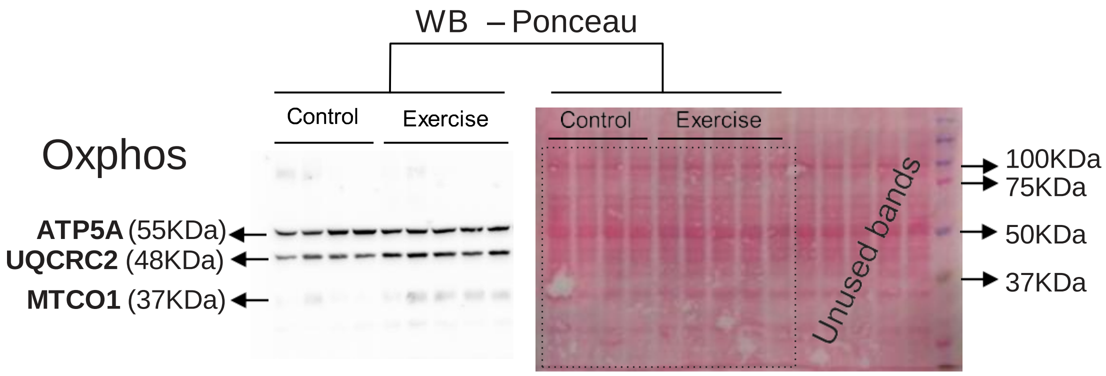

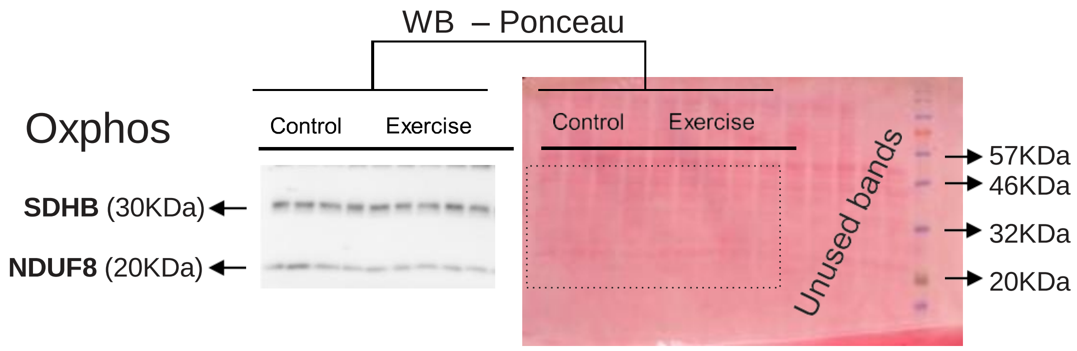

Figure 4G - Membranes, bula and ponceau staining

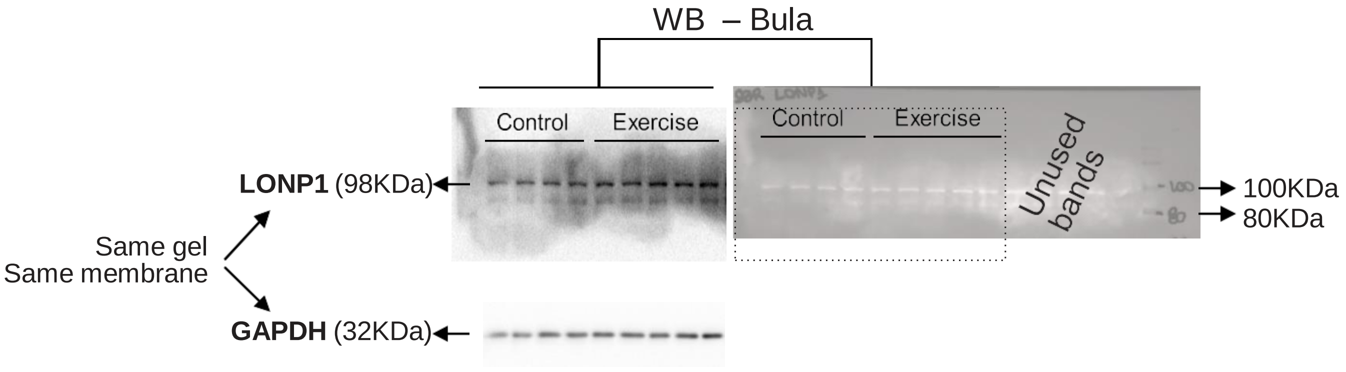

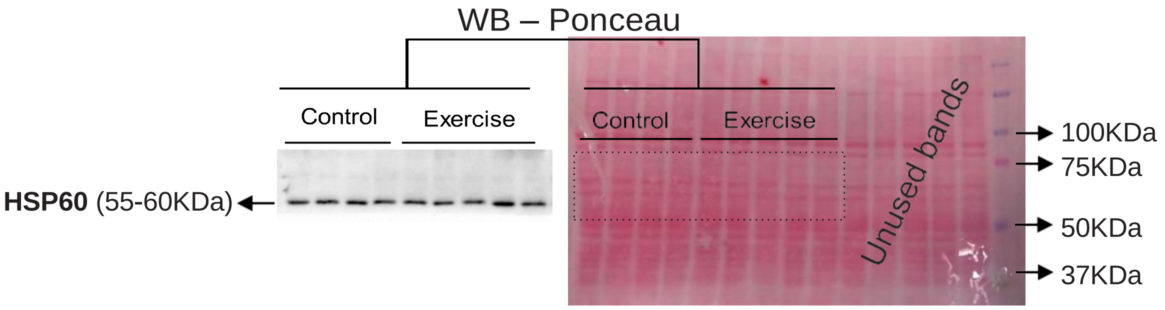

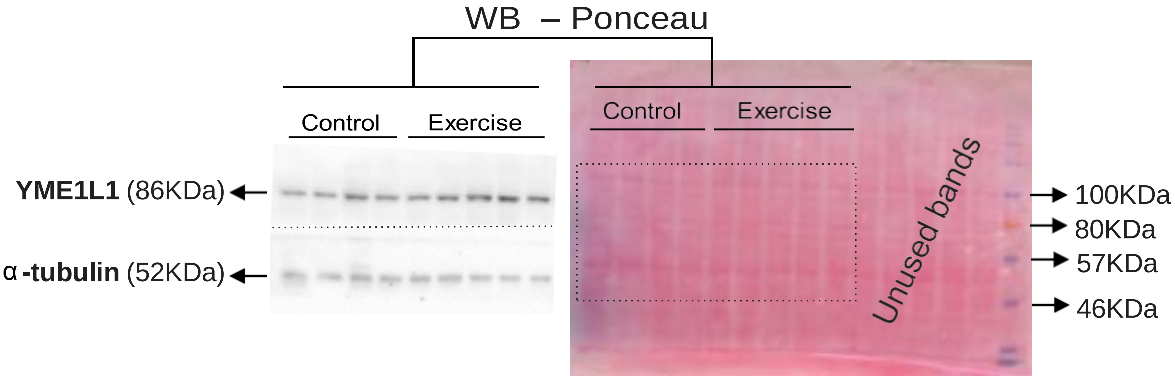

Figure 4H - Membranes and ponceau staining

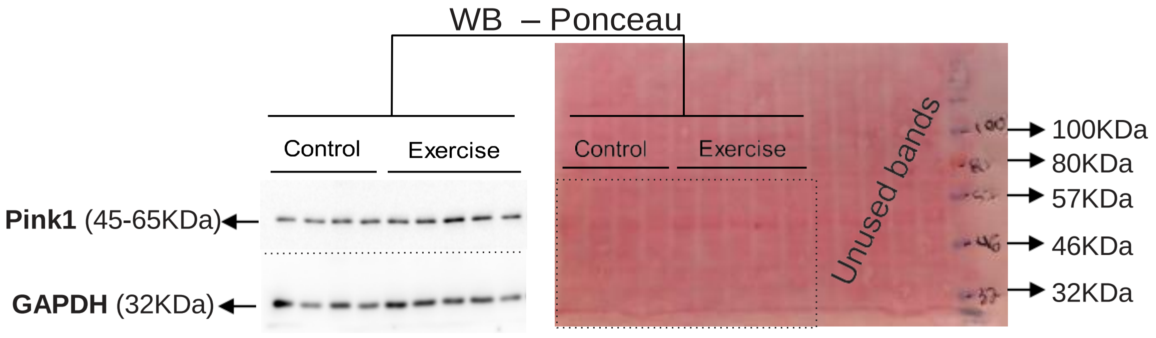

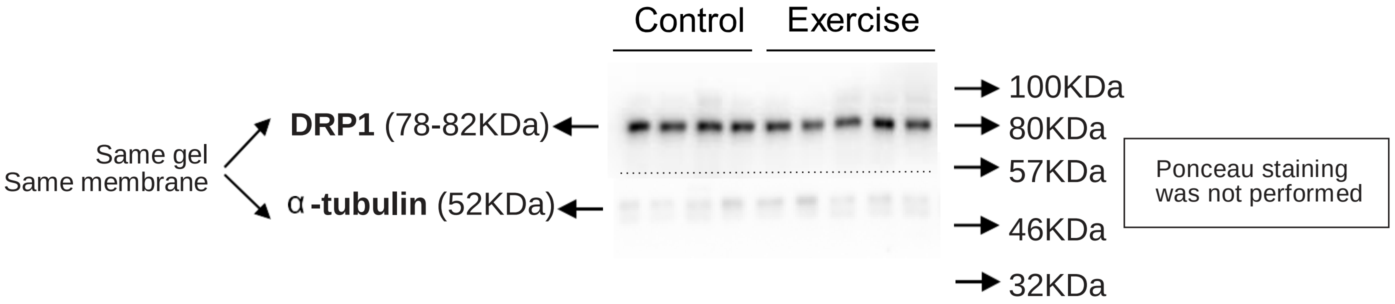

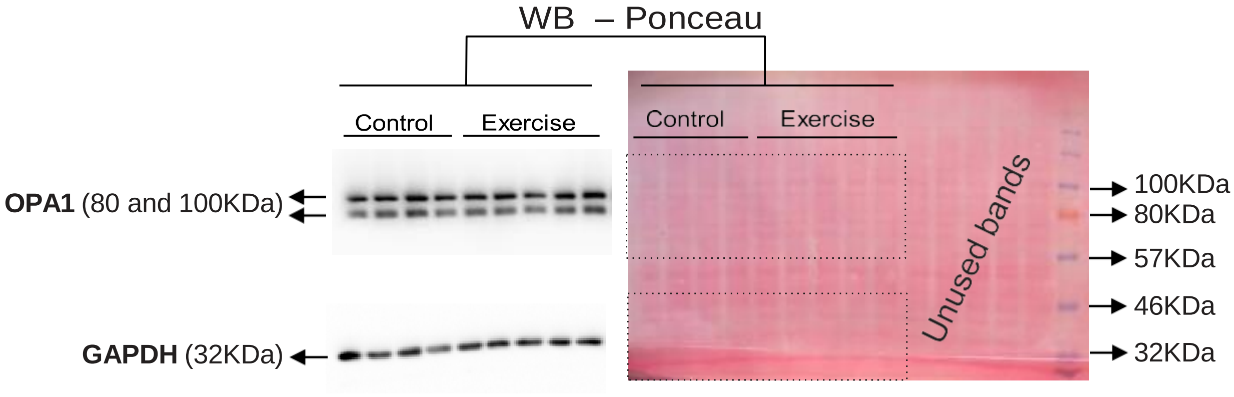

Figure 4I - Membrane and bula.

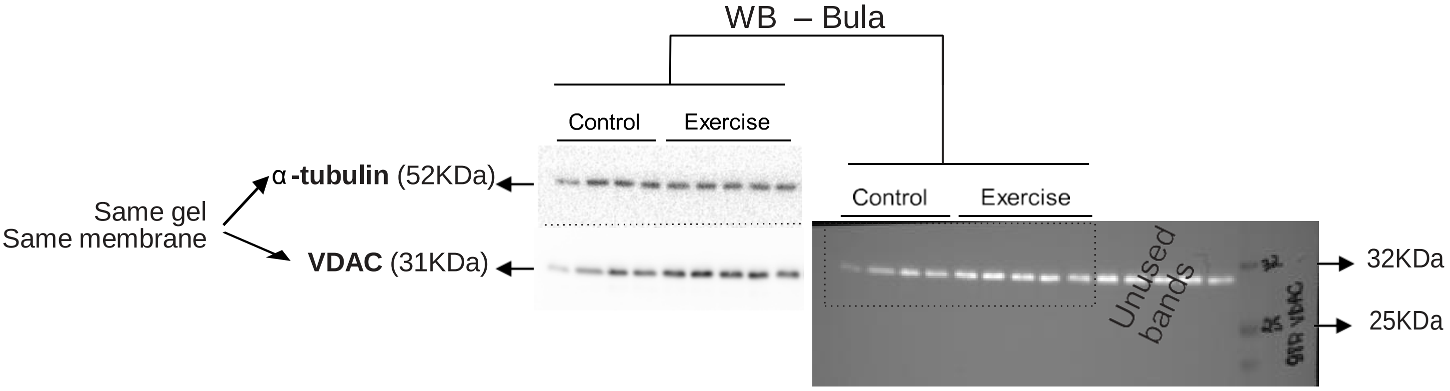

Table 1

PRIMER SEQUENCES		
	**FOWARD**	**REVERSE**
**Mt-Co1**	5’- TCTTTGGGCACCCAGAAGTT -3’	5’- AAGCCTAGAAAGCCAATAGACA-3’
**Mt-Nd1**	5’- AGCCGTAGCCCAAACAATTTCAT -3’	5’- TTATGGCTATGGGTCAGGCTGG -3’
**Atp5a**	5’- CCATTTTGTGCCAGTCGTCC -3’	5’- AAGCATTTTTGGAGACCAGTCC -3’
**Sdha**	5’- ATTCATTGTCTACTTCTCACT -3’	5’- AGGGTTTATTTGGCTTACA-3’
**Lonp1**	5’- AGCCCTATGTTGGCGTCTTC -3’	5’- CCGGCTGATGTGAATCCTTCT -3’
**Npy**	5’- ATACTACTCCGCTCTGCGAC -3’	5’- GAAGGGTCTTCAAGCCTTGTTC -3’
**Pomc**	5’- CCCAGGAACAGCAGCAGTG -3’	5’- GGGGCCTTGGAATGAGAAGAC-3’
**GAPDH**	5'- AGGTCGGTGTGAACGGATTTG -3'	5'- TGTAGACCATGTAGTTGAGGTCA -3'

Table 1. Primers sequences utilized in RT-qPCR for mt-Co1, mt-Nd1, Atp5a, Sdha, Lonp1, Npy, Pomc and GAPDH.

Table 2 (Fig. 1)

Data source: The GeneNetwork at http://www.genenetwork.org

Citations: Please see http://www.genenetwork.org/reference.html

Date : July 25, 2019

Time: 13:56 GMT

Database INIA Hypothalamus Affy MoGene 1.0 ST (Nov10) (n=15)

Symbol	C57BL/6J	DBA/2J	BXD1	BXD11	BXD12	BXD14	BXD24	BXD27	BXD29	BXD31	BXD32	BXD34	BXD39	BXD40	BXD42
Body weight	28,6	25,9	20,7	19,1	23,2	23,2	25,8	23,5	16,9	22	25,3	23,5	18,5	18,5	21,2
mt-Atp6	13,846	13,828	13,917	13,85	13,86	13,72	13,75	13,816	13,9	13,84	13,82	13,866	14,003	13,78	13,755
mt-Co1	14,156	14,178	14,258	14,19	14,22	14,14	14,11	14,14	14,23	14,2	14,12	14,154	14,311	14,19	14,091
mt-Co2	13,688	13,688	13,758	13,7	13,75	13,63	13,61	13,69	13,81	13,69	13,65	13,719	13,858	13,68	13,647
mt-Nd1	14,584	14,614	14,698	14,62	14,63	14,64	14,6	14,563	14,68	14,6	14,58	14,563	14,762	14,64	14,557
mt-Nd3	13,958	13,966	13,986	13,96	13,92	13,91	13,88	13,866	14	13,81	13,89	13,954	14,023	13,9	13,78
mt-Nd4l	14,125	14,113	14,221	14,15	14,16	14,02	14,07	14,104	14,2	14,12	14,08	14,14	14,282	14,14	14,059
mt-Rnr1	14,216	14,228	14,293	14,22	14,29	14,3	14,15	14,176	14,26	14,23	14,21	14,203	14,327	14,22	14,154
mt-Tp	6,518	6,396	6,506	6,368	6,523	6,459	6,589	6,404	6,392	6,535	6,272	6,381	6,545	6,527	6,273
mt-Tq	8,584	8,402	8,354	8,38	8,189	7,093	8,167	8,845	8,162	8,554	8,148	8,762	7,548	7,64	8,977
mt-Ts1	6,315	5,989	5,822	6,276	6,045	5,518	6,599	6,736	6,24	6,554	6,334	6,338	5,957	6,164	6,874
Timm8a1	9,152	9,398	8,724	8,918	8,794	8,976	9,362	8,999	8,838	8,953	9,12	9,012	8,796	8,706	9,073
Mrps22	9,266	9,337	9,088	9,108	9,212	9,133	9,373	9,234	9,067	9,235	9,338	9,224	9,05	9,218	9,288
Acsf2	9,275	9,067	8,945	9,026	9,026	9,062	9,16	9,07	8,907	9,174	9,247	9,213	8,676	9,023	9,114
Acad8	9,084	9,053	8,823	8,956	8,982	8,983	8,952	9,13	8,908	8,979	9,107	9,085	8,823	8,909	9,001
Ndufb2	9,415	9,506	9,061	9,174	9,105	9,046	9,462	9,312	8,958	8,931	9,445	9,37	8,971	9,274	9,344
Uqcc1	9,957	9,911	9,74	9,779	9,864	9,731	9,991	9,93	9,762	9,789	10	9,912	9,667	9,9	9,911
Ndufa6	10,34	10,396	9,796	10,02	9,929	9,824	10,29	10,114	9,806	9,979	10,27	10,084	10,029	10,14	10,237
CYTB	14,323	14,32	14,447	14,37	14,35	14,39	14,3	14,331	14,43	14,36	14,29	14,312	14,492	14,35	14,273
Ndufaf1	9,653	9,564	9,676	9,384	9,557	9,524	9,619	9,4	9,352	9,58	9,596	9,728	9,455	9,448	9,579
Uqcrq	10,282	10,352	9,991	10,11	9,922	9,895	10,51	10,139	9,928	10,04	10,37	10,116	9,91	10,19	10,183
Uqcrc2	7,194	7,054	7,054	6,928	7,058	7,181	7,288	7,078	7,056	7,02	7,07	6,982	6,944	7,05	7,03
Ndufs1	10,482	10,604	10,279	10,28	10,39	10,31	10,41	10,37	10,34	10,15	10,55	10,533	10,206	10,38	10,412
Atp5o	10,901	11,06	10,715	10,91	10,8	10,75	11,13	10,979	10,79	10,87	11,05	10,936	10,7	10,86	10,904
Mrps33	9,758	9,776	9,416	9,586	9,537	9,446	9,907	9,645	9,488	9,569	9,773	9,58	9,301	9,713	9,749
Acat2	9,945	10,084	9,815	9,873	10,01	9,991	10,06	9,878	9,844	9,98	10,14	10,002	9,852	9,972	10,065
Acadl	9,472	9,37	8,876	9,236	9,21	8,646	9,182	9,176	8,947	9,207	9,312	9,242	8,668	9,123	9,134
Uqcr10	9,616	9,66	9,305	9,548	9,346	9,406	9,82	9,625	9,406	9,557	9,612	9,512	9,18	9,544	9,574

Table 2. Animal data of the correlation between total body weight and mRNA levels of nuclear and mitochondrial-related genes in the hypothalamus of 15 strains of BXD mice acquired by the accessible database on Genenetwork (http://www.genenetwork.org).

Table 3 (Fig. 2)

Data source: The GeneNetwork at http://www.genenetwork.org

Citations: Please see http://www.genenetwork.org/reference.html

Date : April 07, 2019

Time : 18:38 GMT

ID 17605 Metabolism, morphology: Body weight loss percentage after exercise, chow diet (6% kCal/fat Harlan.2918), males [%] (EPFL LISP3 Cohort)

Symbol	BW loss	Sdhb	Atp5j	mt-Atp6	mt-Co1	mt-Co2	mt-Nd1	mt-Nd3	mt-Nd4	mt-Rnr1	mt-Tp	mt-Tq	mt-Ts1	Sdha	Ppargc1a
C57BL/6J	3,78	11,137	12,186	8,492	14,13	13,687	14,585	13,93	14,113	14,178	6,436	8,433	6,506	12,385	10,862
DBA/2J	8,54	11,008	12,148	8,289	14,182	13,686	14,593	13,96	14,123	14,227	6,394	8,469	6,02	12,346	10,881
BXD1	-1,77	10,724	11,978	7,878	14,316	13,821	14,734	14,044	14,289	14,307	6,496	8,168	5,54	12,512	10,516
BXD32	2,14	11,082	12,17	8,162	14,123	13,67	14,587	13,963	14,101	14,23	6,301	8,473	6,548	12,451	10,851
BXD39	-0,34	10,866	12,068	8,227	14,311	13,858	14,762	14,023	14,282	14,327	6,545	7,548	5,957	12,366	10,791
BXD40	2,07	11,206	12,16	7,558	14,114	13,594	14,563	13,829	14,036	14,156	6,681	7,927	6,553	12,386	10,706
BXD43	5,90	11,204	12,134	10,348	14,02	13,544	14,471	13,825	13,957	14,159	6,735	9,174	6,946	12,345	10,99
BXD44	7,44	11,092	12,052	8,392	14,01	13,537	14,447	13,789	13,945	14,094	6,397	8,915	6,358	12,168	10,983
BXD45	1,96	10,861	12,105	7,097	13,956	13,432	14,423	13,75	13,911	14,018	6,676	9,54	6,437	12,376	10,777
BXD48	5,50	11,161	12,111	8,255	14,228	13,752	14,632	13,999	14,161	14,242	6,21	8,89	6,734	12,304	10,755
BXD48a	5,01														
BXD49	-5,68	11,101	12,113	9,457	14,138	13,573	14,569	13,764	14,05	14,232	6,632	8,544	6,091	12,322	10,829
BXD50	2,62	10,932	12,107	8,802	14,231	13,811	14,639	13,954	14,201	14,283	6,377	8,953	6,688	12,335	10,991
BXD51	6,46														
BXD53	4,66														
BXD55	4,55														
BXD56	5,09	10,952	12,122	8,427	14,155	13,632	14,588	13,896	14,07	14,211	6,468	8,47	6,32	12,354	10,61
BXD60	2,99	11,213	12,162	8,725	14,019	13,588	14,478	13,835	13,964	14,142	6,437	8,867	6,727	12,401	11,052
BXD61	11,13														
BXD62	-0,78	11,362	12,29	8,587	13,94	13,522	14,423	13,766	13,947	14,111	6,354	8,702	7,158	12,468	10,753
BXD63	5,67														
BXD64	6,38														
BXD65	-0,88	11,018	12,145	7,871	14,085	13,619	14,541	13,857	14,043	14,144	6,335	8,295	6,669	12,4	10,895

**BXD65a**	9,67	10,924	12,201	8,434	14,167	13,671	14,613	13,953	14,118	14,205	6,484	8,254	6,152	12,348	10,867
**BXD65b**	-0,55	10,832	12,064	8,52	14,122	13,655	14,536	13,91	14,075	14,183	6,469	8,465	6,21	12,426	10,828
**BXD66**	0,20														
**BXD67**	7,16														
**BXD68**	17,88	10,96	12,068	8,19	14,236	13,746	14,708	13,994	14,237	14,32	6,609	8,019	5,5	12,428	10,864
**BXD69**	8,23	11,015	12,205	9,72	13,945	13,51	14,413	13,709	13,923	14,044	6,555	8,422	6,745	12,346	10,984
**BXD70**	6,91	10,831	12,208	8,62	14,115	13,676	14,555	13,776	14,089	14,171	6,434	8,879	6,777	12,228	10,902
**BXD71**	14,69	10,942	12,195	9,342	14,203	13,697	14,566	13,927	14,113	14,266	6,411	8,456	6,18	12,287	11,102
**BXD73**	0,38	10,865	12,099	7,59	14,014	13,495	14,465	13,788	13,98	14,074	6,609	8,586	6,263	12,339	10,884
**BXD73a**	7,03	10,956	12,147	8,32	14,059	13,603	14,523	13,812	14,032	14,136	6,499	8,725	6,51	12,394	10,819
**BXD73b**	4,95	10,938	12,11	7,97	13,998	13,512	14,427	13,788	13,957	14,07	6,322	8,901	6,709	12,405	10,838
**BXD75**	9,32	11,018	12,119	8,221	14,29	13,8	14,735	14,016	14,269	14,32	6,396	8,053	5,868	12,397	10,796
**BXD77**	5,68														
**BXD79**	3,73														
**BXD81**	8,45														
**BXD83**	0,14	10,833	12,079	8,464	14,197	13,649	14,601	13,904	14,162	14,27	6,421	8,555	5,919	12,335	10,644
**BXD84**	15,78	11,585	12,482	7,64	14,107	13,565	14,565	13,923	14,033	14,166	6,527	8,753	7,568	12,43	10,626
**BXD85**	0,12	10,829	12,196	8,119	14,065	13,534	14,498	13,691	13,972	14,046	6,525	9,045	6,51	12,235	10,882
**BXD87**	-0,52	10,76	12,066	7,092	14,14	13,591	14,531	13,866	14,067	14,169	6,425	8,525	6,305	12,261	10,826
**BXD89**	4,86	10,898	12,048	8,231	14,162	13,721	14,571	13,955	14,116	14,24	6,411	8,631	6,485	12,321	10,936
**BXD90**	-2,32	10,738	12,003	8,2	14,476	13,971	14,859	14,121	14,446	14,521	6,373	7,664	5,485	12,523	10,517
**BXD95**	5,27	11,056	12,172	8,312	14,112	13,621	14,532	13,877	14,071	14,135	6,214	7,957	6,374	12,236	11,064
**BXD98**	10,92														
**BXD99**	20,31	10,978	12,197	8,71	14,195	13,701	14,63	13,955	14,123	14,233	6,535	8,328	6,591	12,284	10,858
**BXD100**	1,12	11,016	12,049	10,894	14,41	13,914	14,805	14,087	14,369	14,455	6,352	8,576	6,228	12,488	10,644
**BXD101**	3,51	11,045	12,198	8,084	14,085	13,574	14,498	13,821	13,995	14,109	6,348	8,514	6,389	12,152	10,984

Table 3. Animal data of the correlation between body weight loss in response to three weeks of voluntary exercise and mRNA levels of nuclear and mitochondrial-related genes in the hypothalamus of 49 strains of BXD mice acquired by the accessible database on Genenetwork (http://www.genenetwork.org).

Table 4 (Fig.5)

Data source: The GeneNetwork at http://www.genenetwork.org Citations: Please see http://www.genenetwork.org/reference.html Date : March 10, 2019

Time : 00:41 GMT (n=50)

Symbol	mt-Co1	mt-Nd4l	mt-Nd1	CYTB	mt-Co2	mt-Rnr1	mt-Atp6	Npy5r	Foxo1	Npy2r	Npy1r	Hcrtr2	Hcrtr1	Prkar2a	Npy6r
BXD69	13,946	13,944	14,416	14,138	13,479	14,035	13,604	7,975	9,593	9,396	9,028	8,798	8,587	10,57	6,072
BXD102	13,974	13,962	14,465	14,176	13,5	14,051	13,671	8,07	9,67	9,151	9,065	9,008	8,656	10,623	6,08
BXD45	13,986	13,968	14,447	14,212	13,5	14,068	13,598	7,988	9,49	9,118	9,12	8,82	8,504	10,467	5,64
BXD44	13,992	13,951	14,464	14,187	13,52	14,076	13,656	8,052	9,606	9,224	9,12	8,752	8,654	10,656	6,106
BXD60	13,997	13,931	14,442	14,131	13,526	14,082	13,669	8,123	9,532	9,087	8,973	9,105	8,5	10,571	6,016
BXD73	14,017	13,977	14,48	14,192	13,518	14,095	13,622	7,998	9,42	9,041	8,988	8,756	8,483	10,498	5,614
BXD43	14,02	13,957	14,471	14,156	13,544	14,159	13,737	8,096	9,505	9,03	9,137	9,133	8,352	10,697	5,651
BXD73b	14,02	13,977	14,444	14,174	13,554	14,099	13,677	7,985	9,577	9,128	9,152	8,914	8,476	10,584	5,924
BXD62	14,063	14,046	14,531	14,254	13,62	14,18	13,774	7,965	9,303	9,14	8,924	8,929	8,278	10,477	5,286
BXD65	14,071	14,038	14,516	14,255	13,596	14,14	13,755	7,935	9,482	8,986	8,764	8,922	8,392	10,569	5,734
BXD73a	14,072	14,031	14,505	14,22	13,602	14,168	13,741	8,148	9,477	9,131	9,19	9,063	8,507	10,555	5,876
BXD95	14,088	14,039	14,516	14,27	13,6	14,15	13,738	7,962	9,492	9,21	8,796	8,574	8,456	10,599	5,913
BXD42	14,091	14,059	14,557	14,273	13,647	14,154	13,755	8,001	9,467	9,261	9,044	8,98	8,402	10,516	4,904
BXD87	14,105	14,029	14,506	14,259	13,587	14,157	13,754	7,9	9,529	9,142	8,875	8,612	8,588	10,483	5,68
BXD84	14,107	14,033	14,565	14,23	13,565	14,166	13,698	7,996	9,372	8,814	8,886	8,545	8,553	10,152	5,626
BXD24	14,111	14,07	14,602	14,303	13,61	14,145	13,752	7,937	9,217	8,858	8,689	8,888	8,401	10,408	5,516
BXD65b	14,113	14,07	14,522	14,272	13,66	14,161	13,784	7,83	9,486	9,224	8,924	8,82	8,626	10,694	5,718
BXD89	14,114	14,079	14,558	14,302	13,672	14,234	13,796	7,824	9,428	8,935	8,871	8,56	8,509	10,513	5,842
BXD32	14,124	14,084	14,576	14,287	13,649	14,21	13,818	7,87	9,31	8,986	9,022	8,74	8,381	10,521	5,69
BXD85	14,124	14,062	14,572	14,278	13,616	14,174	13,748	7,862	9,466	9,028	8,936	8,461	8,514	10,518	5,746
BXD68	14,135	14,106	14,576	14,324	13,637	14,209	13,82	7,826	9,435	9,03	8,794	8,499	8,439	10,558	5,699
BXD49	14,138	14,05	14,569	14,305	13,573	14,232	13,863	8,106	9,517	8,959	9,02	8,731	8,501	10,387	5,425
BXD27	14,14	14,104	14,563	14,331	13,69	14,176	13,816	7,798	9,294	9,088	8,744	8,702	8,376	10,57	5,693

BXD14	14,141	14,024	14,644	14,385	13,631	14,304	13,715	7,934	9,535	8,719	8,469	8,528	8,386	10,297	5,905
BXD79	14,144	14,088	14,588	14,321	13,685	14,252	13,854	7,931	9,543	9,157	8,594	8,661	8,399	10,479	5,69
BXD34	14,154	14,14	14,563	14,312	13,719	14,203	13,866	7,82	9,42	8,831	8,988	8,784	8,295	10,608	5,404
C57BL/6	14,156	14,125	14,584	14,323	13,688	14,216	13,846	7,928	9,34	9,172	8,944	8,953	8,332	10,407	5,228
B6D2F1	14,157	14,12	14,562	14,307	13,68	14,186	13,822	7,877	9,422	8,976	8,784	8,688	8,337	10,408	5,579
BXD56	14,166	14,096	14,587	14,334	13,679	14,242	13,837	7,804	9,429	9,12	8,785	8,608	8,715	10,456	5,699
BXD101	14,174	14,093	14,596	14,318	13,638	14,198	13,82	7,694	9,336	8,962	8,712	8,418	8,41	10,57	5,848
BXD70	14,175	14,121	14,611	14,319	13,712	14,194	13,777	7,776	9,394	9,032	8,81	8,563	8,619	10,584	5,751
DBA/2J	14,178	14,113	14,614	14,32	13,688	14,228	13,828	7,816	9,31	8,552	8,715	8,588	8,172	10,372	5,568
BXD63	14,181	14,137	14,609	14,354	13,691	14,214	13,833	7,833	9,341	9,077	8,681	8,528	8,441	10,521	5,54
BXD11	14,187	14,146	14,624	14,37	13,7	14,224	13,854	7,805	9,266	8,922	8,77	8,75	8,473	10,476	5,536
BXD75	14,189	14,154	14,646	14,358	13,71	14,218	13,84	7,971	9,334	8,994	8,754	8,369	8,401	10,441	5,542
BXD40	14,19	14,138	14,639	14,345	13,684	14,215	13,781	7,87	9,221	8,876	8,722	8,459	8,485	10,379	5,651
BXD71	14,192	14,128	14,602	14,363	13,704	14,269	13,824	7,694	9,32	8,79	8,692	8,456	8,53	10,36	5,376
BXD31	14,195	14,12	14,595	14,355	13,692	14,23	13,839	7,758	9,348	9,043	8,767	8,457	8,455	10,438	5,599
BXD99	14,204	14,162	14,631	14,354	13,73	14,244	13,86	7,775	9,421	8,939	8,634	8,617	8,45	10,426	5,65
D2B6F1	14,219	14,167	14,622	14,356	13,753	14,28	13,942	7,84	9,274	8,757	8,98	8,79	8,198	10,399	5,191
BXD12	14,224	14,158	14,634	14,352	13,747	14,286	13,857	7,772	9,297	8,817	8,821	8,44	8,521	10,565	5,082
BXD48	14,228	14,161	14,632	14,345	13,752	14,242	13,87	7,748	9,297	8,93	8,662	8,741	8,287	10,526	5,694
BXD29	14,232	14,204	14,682	14,434	13,807	14,256	13,901	7,614	9,314	8,817	8,782	8,687	8,334	10,499	5,376
BXD50	14,24	14,186	14,662	14,402	13,753	14,271	13,885	7,79	9,286	8,919	8,544	8,34	8,359	10,475	5,584
BXD65a	14,249	14,214	14,689	14,417	13,755	14,288	13,878	7,724	9,354	9,004	8,642	8,6	8,506	10,418	5,64
BXD1	14,258	14,221	14,698	14,447	13,758	14,293	13,917	7,734	9,307	8,916	8,712	8,34	8,434	10,416	5,628
BXD83	14,294	14,256	14,716	14,436	13,76	14,354	13,957	7,67	9,334	8,798	8,656	8,394	8,449	10,462	5,732
BXD39	14,311	14,282	14,762	14,492	13,858	14,327	14,003	7,591	9,03	8,459	8,524	8,219	8,16	10,349	5,367
BXD90	14,312	14,292	14,743	14,478	13,836	14,372	14,006	7,709	9,389	8,605	8,692	8,564	8,382	10,471	5,764
BXD100	14,342	14,324	14,737	14,508	13,848	14,392	14,094	7,574	9,1	8,566	8,631	8,674	8,035	10,326	5,432

Table 4. Animal data of the correlation between hypothalamic mitochondrial-DNA gene expression and orexigenic signals in 50 strains of BXD mice acquired by the accessible database on Genenetwork (http://www.genenetwork.org).
